# Pulmonary Hypertension and Downhill Varices: An Incidental Finding

**DOI:** 10.7759/cureus.79385

**Published:** 2025-02-20

**Authors:** Bianca Thakkar, Teresa Da Cunha, Rachael Hagen, Roopjeet Bath

**Affiliations:** 1 Internal Medicine, University of Connecticut Health, Farmington, USA; 2 Gastroenterology and Hepatology, University of Connecticut, Farmington, USA; 3 Internal Medicine, University of Connecticut, Farmington, USA; 4 Gastroenterology and Hepatology, University of Connecticut Health, Farmington, USA

**Keywords:** anemia, gi bleed, melena, pulmonary hypertension, varices

## Abstract

Downhill varices (DV), rare proximal esophageal varices, are typically associated with superior vena cava (SVC) obstruction. We present the case of an 80-year-old male with severe pulmonary hypertension and chronic obstructive pulmonary disease who presented with shortness of breath upon exertion along with new melena and anemia (hemoglobin 5.9 g/dL). Upper endoscopy revealed non-bleeding DV in the proximal esophagus and a vascular lesion on the tongue, with imaging and venography excluding SVC obstruction. Severe pulmonary hypertension was identified via transthoracic echocardiogram (TTE) and right heart catheterization (RHC) as the probable cause of DV via venous backflow and increased central venous pressure. The patient’s anemia improved with supportive care, and no further gastrointestinal bleeding occurred. Management involves identifying and addressing the underlying cause, with endoscopic intervention reserved for active bleeding. This case highlights the importance of recognizing DV as a potential complication of pulmonary hypertension and underscores the need for individualized diagnostic and therapeutic approaches.

## Introduction

Esophageal varices are dilated submucosal esophageal veins connecting the portal and systemic circulation. Compared to distal esophageal varices, downhill varices (DV) are a rare entity and can occur in the middle and proximal esophagus. This condition is distinct from the more common distal esophageal varices, which are typically associated with portal hypertension.

The pathophysiology of downhill varices involves the obstruction of the superior vena cava (SVC), which leads to increased venous pressure in the upper body. This pressure is then transmitted to the esophageal veins, causing them to dilate and form varices [1]. The obstruction of the SVC can be due to various causes, including malignancies, benign tumors, thrombosis, or external compression from structures such as a goiter or mediastinal masses [2]. The symptoms of downhill esophageal varices (DEV) can vary, but the most common presenting symptoms are hematemesis and melena due to upper gastrointestinal bleeding [1]. Incidental DVs are rare; there are only 20 cases reported in the literature presenting as bleeding [3]. 

Management of DEV requires a unique approach compared to distal varices. Endoscopic therapies, such as variceal band ligation, can be used to control acute bleeding, but definitive treatment focuses on relieving the underlying SVC obstruction, often through endovascular procedures like balloon angioplasty or stenting [4]. When there is concern for bleeding, there are options such as endoscopic band ligation, sclerotherapy, or balloon tamponade, which may temporize active DV bleeding [5]. 

Case reports have highlighted various etiologies and management strategies. For instance, a case of DEV secondary to a benign SVC stenosis due to a dialysis catheter was successfully managed with balloon angioplasty [1]. Another case involved a patient with Behcet's disease, where band ligation effectively controlled the bleeding [4]. Additionally, DEV can occur without SVC obstruction, as seen in a case of Castleman's disease, where varices resolved after tumor resection [6]. We describe a case of a patient with a history of chronic obstructive pulmonary disease (COPD) who presented with shortness of breath and melena and was found to have DVs secondary to severe pulmonary hypertension.

This article was previously presented as a meeting abstract poster at the 2024 American College of Gastroenterology (ACG) Annual Scientific Meeting on October 25-30 in Philadelphia, PA.

## Case presentation

An 80-year-old male with a history of chronic obstructive pulmonary disease, severe pulmonary hypertension, heart failure with reduced ejection fraction, and significant prior smoking history presented to the emergency room with dyspnea upon exertion and melena. Patient reported that over the past three months he had worsening dyspnea on exertion. However, over the past two weeks he had increasing dyspnea with activities of daily living (ADLs). He visited an urgent care center where he was prescribed a course of Augmentin and prednisone for presumed COPD exacerbation, following which he felt no improvement. He also reported that for the past several weeks he noticed black stool, but denied any abdominal pain nausea or vomiting. His last dose of coumadin was the morning prior to admission. He had never had an esophagogastroduodenoscopy (EGD) before, but did have a colonoscopy about seven years ago, although this was not in the chart. He noted that he took naproxen once every three weeks. He did not drink alcohol, smoke cigarettes, or use any recreational drugs. He otherwise reported no fevers or chills, no genitourinary symptoms, no chest pain or palpitations, and no recent weight gain or loss. He had no history of gastrointestinal bleeding or illness.

In the emergency department, he had a pulse rate of 57-62, respiratory rate of 20-25, blood pressure of 145/85, and oxygen saturation of 83% on room air. He was placed on 3L by nasal cannula with improvement to 92%. Labs were notable for a white blood cell count of 8.5, hemoglobin of 5.9 g/L from a baseline of 9 g/L, creatinine of 1.3, blood urea nitrogen (BUN) of 24, B-type natriuretic peptide (BNP) of 986, serum troponin of 0.03, lactic acid of 1.8, and magnesium mildly low at 1.6. Viral panel was negative. Chest X-ray showed possible right basilar atelectasis. The patient was given two units of packed red blood cells.

**Figure 1:**
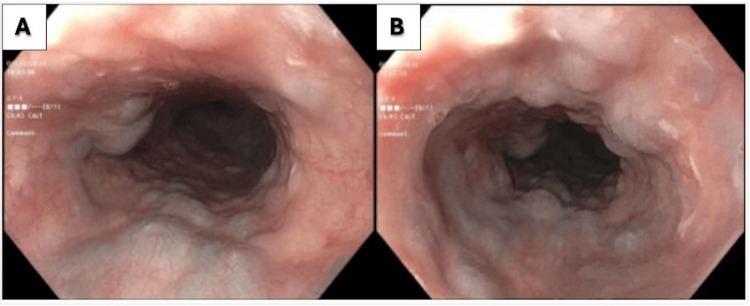
Upper endoscopy with proximal esophageal varices seen as various blebs with a diffuse distribution.

**Figure 2:**
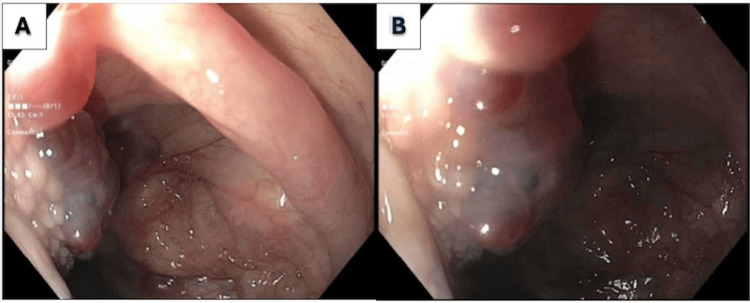
Upper endoscopy with vascular lesion seen at base of tongue, confirmed as sublingual varices on laryngoscopy.

An EGD was performed, which showed non-bleeding varices in the proximal third of the esophagus (Figure 1A, 1B) and a vascular lesion on the base of the tongue (Figure 2A, 2B). A chest venogram to evaluate for SVC obstruction was negative. A flexible laryngoscopy confirmed the presence of sublingual varices.

In the recent past the patient had been diagnosed with pulmonary arterial hypertension (PAH) via transthoracic echocardiography. For that reason, the patient was initiated on specific PAH therapy with sildenafil the week prior. Overall, the patient had been adherent with his medications. Thus, the patient underwent additional diagnostic evaluation with a right heart catheterization. The patient's hemodynamics were consistent with PAH. The patient refused a colonoscopy, and during his hospitalization, he had no further episodes of gastrointestinal bleeding; his anemia improved, and he was discharged. He was instructed to follow up with gastroenterology for further workup of his anemia.

## Discussion

The esophagus is made up of three sections: superior (cervical) drained via the inferior thyroid vein, middle (thoracic) drained via the azygos, hemiazygous, and bronchial veins entering the SVC, and lower esophagus drained via the portal vein. Portal hypertension often translates to “uphill” varices, while “downhill" varices, which are proximal esophageal varices, result from increased pressure or obstruction of the SVC in the distal end of the esophagus causing dilation of the vasculature [1]. The primary symptoms of DEV include hematemesis and melena due to gastrointestinal bleeding. Patients may also present with symptoms related to SVC obstruction, such as facial and upper extremity swelling and dyspnea [4].

The most common cause of DV is SVC syndrome or related SVC obstruction. Benign causes include mediastinal fibrosis, retrosternal goiter, Behcet’s syndrome, chronic obstructive pulmonary disease, or pulmonary hypertension [3]. Malignant causes include bronchial and thyroid carcinomas, lymphomas in the mediastinum, or metastasis [7]. Less commonly, mediastinal tumors, such as thymomas or Castleman’s disease can cause DV via SVC compression [2]. Central venous catheters in hemodialysis patients can lead to SVC stenosis or occlusion. Lastly, large goiters that extend into the mediastinum can cause SVC compression and subsequent DV as well [1].

Initial assessment includes a detailed history and physical examination to identify symptoms of SVC obstruction, such as facial swelling, cyanosis, and distended neck veins [8]. Once found, Computed Tomography (CT) or Magnetic Resonance Imaging (MRI) can be used to determine the underlying cause [3]. They provide detailed anatomical information and help in planning further management [9]. In our case, chest venogram was negative, indicating no obstruction of the SVC. In this case, it is most likely that he developed DV secondary to severe PAH in the setting of severe COPD, as chest and neck imaging was negative for SVC obstruction. PAH is diagnosed by echocardiogram or right heart catheterization and is characterized by an elevated mean pulmonary artery pressure (> 25 mm Hg) and can be associated with increased central venous pressure (CVP). Severe pulmonary hypertension can lead to DV through venous backflow as blood flows from the SVC to the esophageal venous plexus with transmission of elevated intracardiac filling pressures causing congestion [10]. In cases where non-invasive imaging is inconclusive, venography can be performed. This involves injecting contrast material into the venous system to visualize the SVC and its tributaries, confirming the diagnosis of SVC obstruction and the presence of downhill varices [8]. DV are diagnosed via upper endoscopy. Upper endoscopy is performed to directly visualize the esophageal varices and assess their size and risk of bleeding. This is particularly important in patients presenting with gastrointestinal bleeding [8].

There are currently no recommendations for the endoscopic management of DV, but treatment should be aimed at correcting the underlying cause. In cases of thrombosis of the SVC, chemical or mechanical thrombolysis of the clot, venoplasty, and stenting are some options to resolve obstruction [5]. Downhill varices represent 0.1% of all bleedings in the upper gastrointestinal tract, but has been reported rarely in the past, especially in benign causes of DV. Downhill varices are less likely to bleed than uphill varices, which may be because DV have less exposure to gastric acid or because proximal esophageal varices are submucosal as opposed to superficially located distal esophageal varices [7]. Endoscopic variceal band ligation (EVL) is commonly used to control acute bleeding from DEV. This method has been shown to be effective in temporizing bleeding episodes [4]. The cornerstone of definitive treatment is the relief of the underlying SVC obstruction. This can be achieved through endovascular procedures such as balloon angioplasty or stenting of the SVC. In cases where the underlying cause is a tumor, surgical resection may be necessary [4,10]. Management of coagulopathy and hemodynamic stabilization are critical during acute bleeding episodes. This includes transfusions and correction of clotting abnormalities [4].

## Conclusions

In conclusion, gastroenterologists should be familiar with DV and, when incidentally found, do a full workup for relevant underlying causes. Diagnostic workup changes based upon the patient and clinical presentation, but can include endoscopy/endosonography, duplex sonography of the veins of the throat, transesophageal echocardiography, sonography of the thyroid/throat, computerized tomography of the thorax/throat, magnetic resonance imaging. Chest and neck imaging should be ordered to evaluate for SVC obstruction and, if negative, right heart catheterization should be pursued to evaluate for pulmonary hypertension as a cause of DV if clinically appropriate. Prognosis of primary DV is very good but can be complicated by bleeding when associated with superior vena cava syndrome. Treatment is aimed at correcting the underlying cause and temporizing any bleeding with band ligation and sclerotherapy. 
